# Dynamic Factors for Transmitter Release at Small Presynaptic Boutons Revealed by Direct Patch-Clamp Recordings

**DOI:** 10.3389/fncel.2019.00269

**Published:** 2019-06-12

**Authors:** Shin-ya Kawaguchi

**Affiliations:** ^1^Society-Academia Collaboration for Innovation, Kyoto University, Kyoto, Japan; ^2^Department of Biophysics, Graduate School of Science, Kyoto University, Kyoto, Japan; ^3^Institute for Advanced Study, Kyoto University, Kyoto, Japan

**Keywords:** axon, patch-clamp, transmitter release, presynaptic terminal, action potential, short-term plasticity

## Abstract

Small size of an axon and presynaptic structures have hindered direct functional analysis of axonal signaling and transmitter release at presynaptic boutons in the central nervous system. However, recent technical advances in subcellular patch-clamp recordings and in fluorescent imagings are shedding light on the dynamic nature of axonal and presynaptic mechanisms. Here I summarize the functional design of an axon and presynaptic boutons, such as diversity and activity-dependent changes of action potential (AP) waveforms, Ca^2+^ influx, and kinetics of transmitter release, revealed by the technical *tour de force* of direct patch-clamp recordings and the leading-edge fluorescent imagings. I highlight the critical factors for dynamic modulation of transmitter release and presynaptic short-term plasticity.

## Introduction

Direct patch-clamp recording from an axonal compartment tells a lot about the axon physiology and mechanisms of synaptic transmission. Unfortunately an axon and presynaptic terminals are usually too small to apply patch-clamp technique, except for unusually large structures, such as synapses of calyx of Held in the auditory brainstem and presynaptic boutons of hippocampal mossy fiber (Forsythe, 1994; Borst et al., 1995; Geiger and Jonas, 2000). Most conventional synapses in the central nervous system have much smaller presynaptic boutons (∼1 μm) containing only 1–2 active zones, from which direct recording has been almost impossible to perform. However, in this decade remarkable technical advances have been accomplished in recording even from conventional small presynaptic structures in primary culture system, such as boutons of hippocampal pyramidal cells (∼1 μm, Novak et al., 2013), cerebellar Purkinje cell (PC) axon terminals (∼2–3 μm, Kawaguchi and Sakaba, 2015), and cerebellar granule cell (GC) axon varicosities (∼1 μm, Kawaguchi and Sakaba, 2017). These advancements have provided detailed information about the mechanisms of presynaptic function, such as dynamics of presynaptic membrane excitability, Ca^2+^ influx and buffering, pool size of readily releasable synaptic vesicles, and functional coupling between Ca^2+^ and release machinery. In this article, I like to overview these recently clarified presynaptic mechanisms for transmitter release, particularly focusing on small synapses.

## Factors to Determine Presynaptic Transmitter Release

**FIGURE 1:**
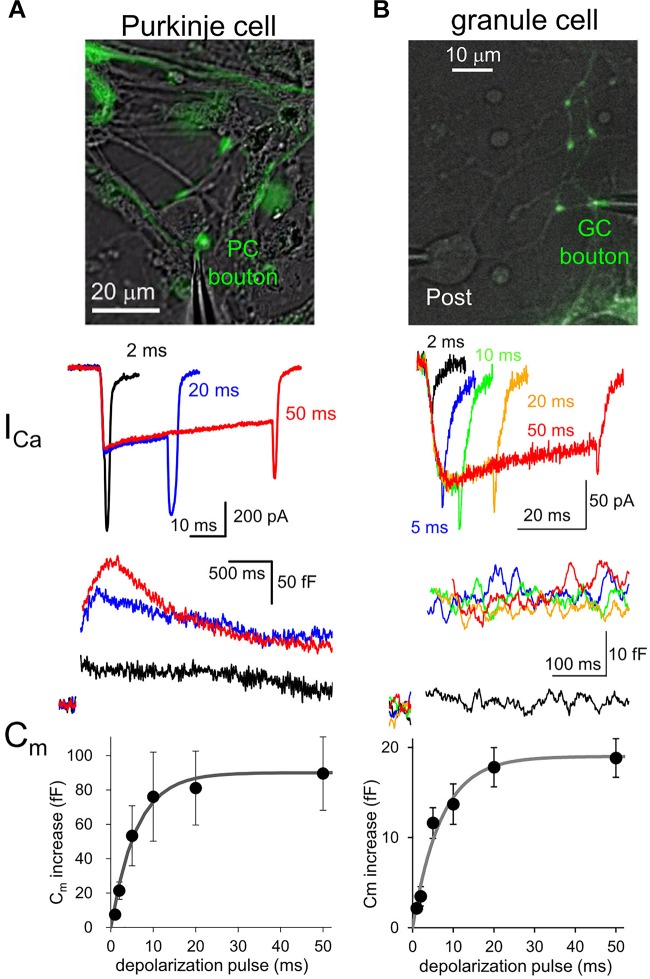
Presynaptic transmitter releases studied by direct patch clamp recordings. **(A**,**B)** Patch-clamp recordings from a EGFP-labeled axon varicosity of a cerebellar PC **(A)** or of GC **(B)**, adapted from Kawaguchi and Sakaba (2015, 2017), respectively, with permission from Elsevier. Top, images of EGFP-labeled axon terminals of each neuron. Middle, presynaptic Ca^2+^ currents (I_Ca_) upon different durations of depolarization pulses to 0 mV. Bottom, presynaptic Cm increase triggered by Ca^2+^ currents indicated in the middle. Average Cm increases plotted against duration of depolarization pulses are also shown.

Fluorescent labeling of axons and presynaptic boutons makes it possible to precisely position a thin tip of glass pipette at a small structure for patch-clamp recordings. Voltage-clamp of an axon terminal allows to study quantitative relationship between the Ca^2+^ influx to a presynaptic bouton and the amount of exocytosis of synaptic vesicles (Figure 1). For example, a long-duration of depolarization pulse applied to a bouton triggers fusion of almost all release-ready synaptic vesicles to the cytoplasmic membrane, resulting in an increase of surface membrane area size which can be recorded as a sudden jump of membrane capacitance (Cm) (Lindau and Neher, 1988; Figure 1). The amount of Cm increase, that is, the number of synaptic vesicles in readily releasable pool (RRP) changes depending on the type of synapses. For example, calyx of Held synapse (diameter, ∼10 μm) shows about 400 fF of Cm increase upon a large Ca^2+^ influx as ∼2 nA upon a presynaptic depolarization pulse to 0 mV (Sun and Wu, 2001). On the other hand, ∼100 fF of Cm increase is caused by ∼200 pA of Ca^2+^ currents at mossy fiber boutons in hippocampus (∼5 μm) and those in the cerebellum (∼6 μm) (Hallermann et al., 2003; Delvendahl et al., 2013). Recently, an inhibitory neuronal presynaptic terminal, a PC bouton with a size of 2∼3 μm was patched, and a Cm increase of 80 fF was observed upon depolarization-induced Ca^2+^ influx of ∼300 pA (Kawaguchi and Sakaba, 2015; Figure 1). Thus, given that a single synaptic vesicle has a Cm of ∼ 0.1 fF, about 1,000 synaptic vesicles are exocytosed from a single presynaptic bouton of a mossy fiber or a PC within several hundred milliseconds. On the other hand, a much smaller conventional presynaptic structure (∼1 μm), an axon varicosity of a cerebellar GC, exhibited a ∼20 fF of Cm increase with ∼60 pA of Ca^2+^ currents upon the identical depolarization pulse (Kawaguchi and Sakaba, 2017; Figure 1). Thus, even at a small axon terminal about 200 synaptic vesicles rapidly undergo fusion, which surprisingly corresponds to one third of total synaptic vesicles in a single bouton, based on the estimation by an electron microscopic analysis (Xu-Friedman et al., 2001). It should be noted that the quality of patch-clamp recording (i.e., physical access to the cytosol reflected by the series resistance) is critical for precise activation and measurement (in the extent and time course) of Ca^2+^ currents and vesicular release. These measurements of presynaptic Cm increase upon strong Ca^2+^ influx suggests that substantial number of synaptic vesicles rapidly undergo exocytosis at most synapses irrespective of their being excitatory or inhibitory.

Interestingly, comparison of various neuronal terminals implies a relationship that the maximal Cm increase and presynaptic Ca^2+^ current amplitude likely depend on the first to second power of presynaptic diameter. Thus, we are tempted to assume that the RRP size estimated by Cm measurement simply reflects the surface area size of presynaptic structure, implying that the release machinery downstream of activation by Ca^2+^ may operate in a similar manner at various neuronal boutons. However, it should be noted that the vesicular release estimated by Cm increases upon depolarization for tens of ms sometimes reflects the fusion of multiple states of synaptic vesicles, including those already primed at and those loosely coupled to the release sites (Sakaba and Neher, 2001), and even newly recruited ones at an extremely rapid rate (∼ several ms) (Saviane and Silver, 2006; Kawaguchi and Sakaba, 2017). Recent elegant glutamate imaging coupled with super-resolution analysis of active zone proteins and electron microscopic analysis suggested that the exact number of release sites, which corresponds to synaptic vesicles maximally ready for immediate exocytosis upon Ca^2+^ influx, is defined by the number of protein complex clusters consisting of Ca^2+^ channels, and some active zone protein like Munc-13 (Miki et al., 2017; Sakamoto et al., 2018). Thus, the molecular organizations of presynaptic active zone and the vesicular recruitment mechanisms toward empty release sites determine the strength and sustainability of presynaptic transmitter release.

**FIGURE 2:**
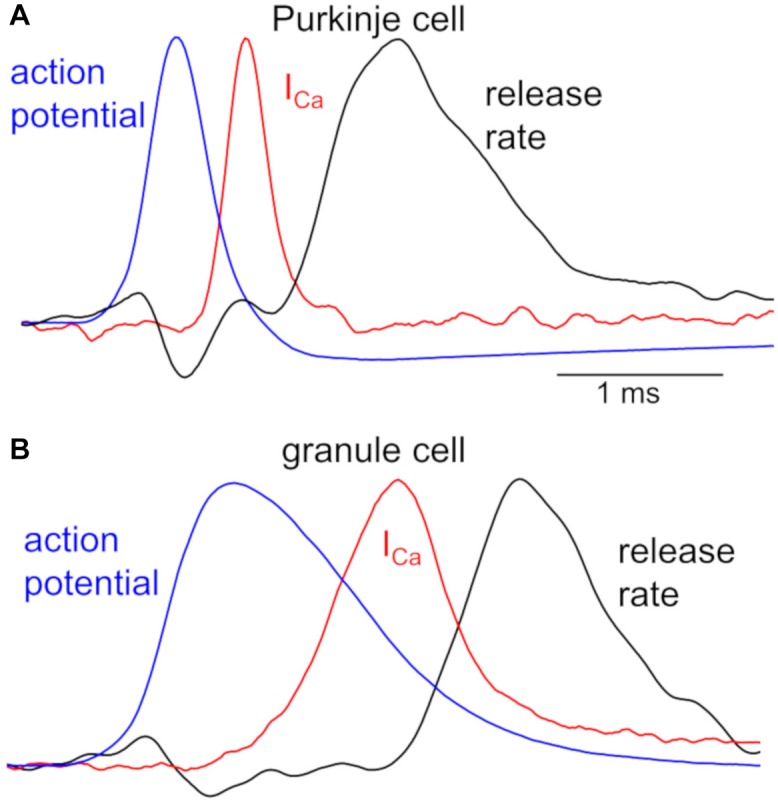
Kinetics of APs, Ca^2+^ influx, and transmitter release in a PC and GC bouton. Peak-scaled representative waveforms of APs, presynaptic Ca^2+^ currents (I_Ca_), and transmitter release rate obtained by simultaneous recordings from a representative pair of presynaptic bouton (**A**, PC; **B**, GC) and postsynaptic cell. Spontaneous APs (blue), recorded from an EGFP-labeled axon varicosity of a PC (**A**, peak of +30 mV) and of a GC (**B**, peak of +37 mV), were used as voltage commands for stimulation to the voltage-clamped bouton, and presynaptic I_Ca_ (red, average of 5 traces from a pair) and postsynaptic currents were simultaneously recorded. Time courses of transmitter release rate (black, average of 5 traces recorded from a pair) were calculated by the deconvolution of the postsynaptic responses evoked by the presynaptic I_Ca_, based on the miniature postsynaptic responses. Presynaptic recordings from a bouton of PC axon and that of GC axon had similar recording conditions, precluding the possibility of the distinct kinetics being due to the quality of patch-clamp recordings. All traces are adapted from Kawaguchi and Sakaba (2015, 2017) with permission from Elsevier.

Different neuronal terminals show diversity in the process from Ca^2+^ channel activation to triggering of vesicular release. First, Ca^2+^ current is rapidly activated at a PC bouton (Kawaguchi and Sakaba, 2015) like a calyx of Held synapse (Borst et al., 1995), whereas a GC bouton exhibits slow activation of Ca^2+^ current upon the identical depolarization pulses (Kawaguchi and Sakaba, 2017; Figure 1). In addition, the Ca^2+^-release coupling estimated by the sensitivity to Ca^2+^ chelators like EGTA varies in different neuronal boutons: a PC bouton shows tight coupling with low sensitivity to EGTA (Díaz-Rojas et al., 2015; Kawaguchi and Sakaba, 2015), as in other inhibitory interneurons (Bucurenciu et al., 2008; Eggermann et al., 2012); a GC bouton shows loose coupling with high EGTA sensitivity (Kawaguchi and Sakaba, 2017), in a similar manner to hippocampal mossy fiber boutons (Vyleta and Jonas, 2014). Furthermore, even action potential (AP) waveforms are quite different in axon terminals of PCs and GCs (see Figure 2).

Simultaneous recordings both from a presynaptic bouton and a postsynaptic cell provide more quantitative information about synaptic transmission (Sakaba and Neher, 2001; Sun and Wu, 2001). Figure 2 illustrates time courses of presynaptic APs and Ca^2+^ influx recorded from a PC or GC bouton, together with the kinetics of vesicular release estimated from postsynaptic responses (Kawaguchi and Sakaba, 2015, 2017). The remarkably different AP waveforms, when applied to the voltage-clamped bouton as voltage commands, cause clearly distinct kinetics of Ca^2+^ influxes. This difference in Ca^2+^ influx might be partly due to the slower activation of Ca^2+^ channels in a GC than in a PC bouton (see Figure 1), in addition to the distinct AP kinetics (Figure 2). Furthermore, the initiation of transmitter release delays more in a GC axon varicosity after the Ca^2+^ influx (∼500 μs after the time of half-maximal I_Ca_) than in a PC bouton (∼300 μs), in spite that once started the release kinetics are similar (Figure 2). This distinct delay of release onset may partially reflect the different Ca^2+^-release coupling: tight coupling at a PC bouton, whereas loose coupling in a GC bouton. On the other hand, similar time course of release rate by itself implies that release machinery may operate in a similar manner at these neurons. Thus, AP waveforms, the resultant activation of Ca^2+^ channels, and the Ca^2+^-driven activation of release machinery, seem to change depending on the neuronal type, and likely become as key factors to impact the transmitter release. Interestingly, recent imaging studies demonstrated that Ca^2+^-release coupling becomes tighter after the chemical induction of presynaptic long-term potentiation (LTP) in hippocampal mossy fiber boutons (Midorikawa and Sakaba, 2017).

## Digital and Analog Signaling in an Axon

Traditionally AP has been regarded as an all-or-none type of digital signal, reliably conveying information toward terminals in the central nervous system of vertebrates. However, direct recordings of APs by axonal patch-clamp methods and fluorescent imagings of membrane potential with a genetically encoded or chemical voltage indicator, have demonstrated activity-and/or location-dependent dynamic changes of AP waveforms in axon terminals (Kole et al., 2007; Hoppa et al., 2014; Kawaguchi and Sakaba, 2015; Rowan et al., 2016). In addition, sub-threshold electrical signals affect AP waveforms by modulating axonal K^+^ channels and/or by passively propagating into an axon over hundreds μm even around distal regions (Pouzat and Marty, 1999; Alle and Geiger, 2006; Shu et al., 2006; Kole et al., 2007; Paradiso and Wu, 2009; Trigo et al., 2010; Pugh and Jahr, 2013; Zbili et al., 2016), which results in the modulation of transmitter release from presynaptic terminals (Rama et al., 2015; Rowan and Christie, 2017; Zorrilla de San Martin et al., 2017). Thus, the axonal AP signaling is modified in a manner like a hybrid of analog and digital transmission (Clark and Häusser, 2006; Debanne et al., 2011).

Because all Ca^2+^ channels at a presynaptic structure are not necessarily activated by individual APs, change of an AP waveform potentially impacts the Ca^2+^ influx into presynaptic cytoplasm, leading to modulation of transmitter release. In a cerebellar PC, high frequency AP firing results in attenuation of AP amplitude at a bouton, decreasing peak Ca^2+^ influx, and hence synaptic outputs (Kawaguchi and Sakaba, 2015). On the other hand, at hippocampal mossy fiber synapses, activity-dependent attenuation of AP is accompanied with the slower decay and hence causes larger net influx of Ca^2+^, leading to augmented transmission (Geiger and Jonas, 2000). Distinct Ca^2+^-release coupling in PC boutons and mossy fiber boutons might be partially responsible for the apparently opposite effects of activity-dependent AP changes on transmitter release (Vyleta and Jonas, 2014; Kawaguchi and Sakaba, 2015). The synapses with tighter Ca^2+^-release coupling like PC boutons would show steeper dependence of transmitter release on the number of activated Ca^2+^ channels rather than the total amount of Ca^2+^ influx. Then, the AP amplitude controlling the maximal number of activated Ca^2+^ channels could be more influential for the release in such tightly coupled synapses than the decay time course affecting the duration of Ca^2+^ influx. In contrast, the total Ca^2+^ influx would more preferentially control transmitter release in loosely coupled synapses. Thus, the impact of AP waveform modulation is determined how the synapse is functionally designed at the molecular level.

The presence of receptors for neurotransmitters in an axon has been demonstrated at various brain region and influences axonal excitability and neurotransmitter release (Debanne et al., 2011). Because of the substantial passive traveling of electrical signals in an axon, even a limited number of receptors in individual boutons could cooperatively contribute to changing the local membrane potential in an axon. For example, the axonal GABA_A_Rs have been reported at various excitatory and inhibitory neurons, such as those in calyx of Held, posterior pituitary, cerebral cortex, hippocampus, and cerebellum (MacDermott et al., 1999; Trigo et al., 2008; Zorrilla de San Martin et al., 2017). Axonal GABA_A_Rs may monitor the level of GABAergic inhibition around the postsynaptic target, locally adjusting the amount GABA release. Perforated patch-clamp technique using gramicidin clarified the reversal potential of GABA-mediated current (E_GABA_) as ∼-50 mV in a terminal of the calyx of Held synapse (Price and Trussell, 2006) and -45 mV in a PC bouton (Zorrilla de San Martin et al., 2017). Because of the depolarized E_GABA_, presynaptic GABA_A_R activation depolarizes presynaptic boutons, facilitating Ca^2+^ influx upon the subsequent AP arrival and hence synaptic transmission. In contrast, axonal GABA_A_Rs also exert inhibitory effects in different neurons (Xia et al., 2014).

## Short-Term Plasticity

Activity-dependent short-term plasticity of synaptic transmission lasting for milliseconds to minutes is an important element in the neuronal computation (Abbott and Regehr, 2004; Regehr, 2012). A lot of central synapses exhibit increase or decrease of strength, termed short-term synaptic facilitation, or depression, respectively, upon repetitive activity at short time intervals (Zucker and Regehr, 2002; Fioravante and Regehr, 2011). Dynamic changes of presynaptic Ca^2+^ concentration and downstream processes of transmitter release mediate short-term plasticity. Residual Ca^2+^ hypothesis is the simplest candidate mechanism for facilitation (Katz and Miledi, 1968). Temporal summation of residual Ca^2+^ remaining in the cytoplasm after the first AP was suggested to facilitate the following AP-triggered transmitter release based on the ∼third power Ca^2+^-dependence of release (Neher and Sakaba, 2008). However, the residual Ca^2+^ increase is too small compared with the local Ca^2+^ to explain synaptic facilitation in most cases.

P/Q-type of Ca^2+^ channels, which predominantly mediates transmitter release at many synapses, exhibit remarkable facilitation of its current in a Ca^2+^-dependent manner, resulting in facilitation of transmitter release (Catterall and Few, 2008; Ben-Johny and Yue, 2014). The association of Ca^2+^ to C-lobe of calmodulin, which is basically bound with the P/Q-type channels, is thought to rapidly augment the Ca^2+^ current by increasing the probability of channel opening. Exogenous expression of mutant Ca^2+^ channels in superior cervical ganglion neurons in culture demonstrated a tight correlation between facilitation of Ca^2+^ current and that of synaptic transmission (Mochida et al., 2008). Paired recordings from pre- and postsynaptic structures of a PC-PC pair showed that facilitation is almost exclusively mediated by the Ca^2+^ current facilitation (Díaz-Rojas et al., 2015). Similar Ca^2+^-dependent facilitation of presynaptic Ca^2+^ influx takes place at glutamatergic boutons in the calyx of Held, which partly contributes to facilitation of synaptic transmission (Felmy et al., 2003; Müller et al., 2008; Hori and Takahashi, 2009). However, recently it was reported that a mutant mice lacking Ca^2+^-facilitation of P-type channels still exhibit short-term facilitation at many synapses in a physiological condition, suggesting just a minor contribution of Ca^2+^-current facilitation to short-term synaptic facilitation (Weyrer et al., 2019).

Another candidate mechanism for synaptic facilitation is the Ca^2+^ buffer saturation hypothesis. If Ca^2+^ buffer molecules remain occupied by Ca^2+^ that entered into a bouton upon the first AP, cytoplasmic free Ca^2+^ becomes more abundant upon the following APs, and leading to larger transmitter release. Indeed, calbindin contributes to the facilitation at hippocampal mossy fiber-CA3 excitatory synapses and inhibitory synapses in the cerebral cortex (Blatow et al., 2003). The buffer saturation model relies on the loose coupling between Ca^2+^ and release machinery (Neher, 1998; Rozov et al., 2001; Vyleta and Jonas, 2014). Recently, direct recordings from a cerebellar GC axon varicosity demonstrated that Ca^2+^-release coupling is indeed loose there, and facilitation depends on the presence of low concentration of fast Ca^2+^ buffer in the terminal (Kawaguchi and Sakaba, 2017), in line with the Ca^2+^ buffer saturation model.

The next hypothesis for short-term facilitation is the Ca^2+^-dependent facilitation of release sensors (Regehr, 2012). Recent accumulating results indicate the critical role of Syt7 as such a facilitation sensor at various synapses (Jackman et al., 2016; Turecek and Regehr, 2018). The mechanism how Syt7 contributes to facilitation of transmitter release remains to be clarified in the future. However, it should be noted that some facilitation remains even with the genetic ablation of Syt7, implying that different combination of several facilitation mechanisms described above would operate together at distinct synapses.

In addition to facilitation, short-term depression has also been studied, and the RRP depletion is thought to be the major mechanism (Zucker and Regehr, 2002; Fioravante and Regehr, 2011). Other mechanisms, such as Ca^2+^ current inactivation and AP conduction failure at axonal branches (Brody and Yue, 2000; Xu and Wu, 2005) and inactivation of release sites (Neher and Sakaba, 2008; Pan and Zucker, 2009), could also contribute. GABAergic synapses on deep cerebellar nuclei neurons innervated from PCs also exhibit short-term depression upon high frequency activation (Telgkamp and Raman, 2002; Pedroarena and Schwarz, 2003). Recently, direct axonal patch-clamp recordings together with fluorescent imaging of synapto-pHluorin demonstrated frequency-dependent attenuation of AP propagation from axonal tract toward the terminals in PCs (Kawaguchi and Sakaba, 2015). Smaller number of Ca^2+^ channels activated by a smaller AP is responsible for the depression of transmitter release at high-frequency activation in a PC bouton. Importantly, the AP conduction faithfulness seems to increase in PC axons along with the development, resulting in smaller frequency-dependent depression at this synapse in mature animals (Turecek et al., 2016).

## Conclusion and Future Direction

As overviewed here, recent technical advances to study axon physiology are shedding light on rich computational ability of an axon affecting synaptic outputs, rather than the classical view of an axon as a simple reliable digital signal conductor. Transmitter release from a presynaptic bouton is dynamically controlled by a variety of activity-dependent modulations of critical factors, such as AP waveforms, Ca^2+^ channel activation, and Ca^2+^-triggered release processes based on fine molecular organizations of release sites and vesicular replenishment systems. Different combinations of these dynamic elements would define the neuronal type-specific functional design of synapses and their short- and/or long-term plasticity. Rapid expansion of techniques for direct recording of axonal signaling with subcellular patch-clamp methods and/or fluorescent imagings is going to clarify neuron-specific and common functional designs of a variety of CNS synapses in the near future, which enriches our understanding of neuronal circuitry.

## Author Contributions

S-yK wrote the manuscript and prepared the figures.

## Conflict of Interest Statement

The author declares that the research was conducted in the absence of any commercial or financial relationships that could be construed as a potential conflict of interest.
